# Lifestyle Interventions and Prevention of Suicide

**DOI:** 10.3389/fpsyt.2018.00567

**Published:** 2018-11-06

**Authors:** Isabella Berardelli, Valentina Corigliano, Michael Hawkins, Anna Comparelli, Denise Erbuto, Maurizio Pompili

**Affiliations:** ^1^Department of Neurosciences, Mental Health and Sensory Organs, Suicide Prevention Center, Sant'Andrea Hospital, Sapienza University of Rome, Rome, Italy; ^2^Department of Psychiatry, University of Toronto, Toronto, ON, Canada

**Keywords:** suicide, suicide attempts, suicidal thoughts, lifestyle behavior, lifestyle intervention, suicide prevention

## Abstract

Over the past years, there has been a growing interest in the association between lifestyle psychosocial interventions, severe mental illness, and suicide risk. Patients with severe mental disorders have higher mortality rates, poor health states, and higher suicide risk compared to the general population. Lifestyle behaviors are amenable to change through the adoption of specific psychosocial interventions, and several approaches have been promoted. The current article provides a comprehensive review of the literature on lifestyle interventions, mental health, and suicide risk in the general population and in patients with psychiatric disorders. For this purpose, we investigated lifestyle behaviors and lifestyle interventions in three different age groups: adolescents, young adults, and the elderly. Several lifestyle behaviors including cigarette smoking, alcohol use, and sedentary lifestyle are associated with suicide risk in all age groups. In adolescents, growing attention has emerged on the association between suicide risk and internet addiction, cyberbullying and scholastic and family difficulties. In adults, psychiatric symptoms, substance and alcohol abuse, weight, and occupational difficulties seems to have a significant role in suicide risk. Finally, in the elderly, the presence of an organic disease and poor social support are associated with an increased risk of suicide attempt. Several factors may explain the association between lifestyle behaviors and suicide. First, many studies have reported that some lifestyle behaviors and its consequences (sedentary lifestyle, cigarette smoking underweight, obesity) are associated with cardiometabolic risk factors and with poor mental health. Second, several lifestyle behaviors may encourage social isolation, limiting the development of social networks, and remove individuals from social interactions; increasing their risk of mental health problems and suicide.

## Introduction

Several well-established psychosocial factors and lifestyle behaviors including life satisfaction, economic adversities, family environment, major life and occupational stressor(s), substance and alcohol abuse, medical conditions, cigarette smoking, being sedentary, underweight or obese, and diet are considered prominent factors in the development of severe mental disorders and suicide risk. Psychiatric patients, including patients affected by schizophrenia, bipolar disorder, schizoaffective disorder and depressive disorder, have higher mortality rates and higher suicide risk compared to the general population (1, 2). Increased mortality is probably also due, besides psychiatric impairment and psychotropic medication use, to unhealthy lifestyle behaviors including poor diet, excessive smoking, alcohol use, and lack of exercise. These unhealthy lifestyle behaviors can increase the development of many physical diseases including overweight, obesity, metabolic syndrome, diabetes, cardiovascular disease, cerebrovascular disease, hepatitis, amongst other medical conditions (3, 4).

Suicide has been described as a multi-faceted construct in relation to psychiatric symptoms, medical conditions, and lifestyle risk factors (5–7). Moreover, lifestyle behaviors are involved in the pathogenesis of medical and severe psychiatric disorders such as depression and psychosis (8, 9), increasing the risk of suicide (10).

Recently, considerable attention has been paid to the concept of life satisfaction and well-being as psychosocial indicators of mental health (11). It has been hypothesized (12, 13) that life satisfaction and meaning in life can help people overcome adversity. Factors associated with optimal well-being and life satisfaction included prudent lifestyle behaviors such as healthy eating, adequate sleep, physical activity, avoiding tobacco, and limiting alcohol consumption (14). Few studies have revealed that suicide ideation is related to psychiatric symptomatology, lifestyle behavior, and lower life satisfaction (15, 16). Koivumaa-Honkanen et al. (17) in a study of 29,067 Finnish adults found that unhappiness was associated with an increased risk of suicide. This group also reported that unhappiness was associated with older age, male gender, sickness, living alone, smoking, heavy alcohol consumption, physical inactivity, and belonging to intermediate social class.

In the past few decades, a lot of progress has been made to identify public health strategies for the prevention of mental health and suicide. Although the approach to suicide has always involved clinical aspects, especially the assessment and the treatment of depression (18), it seems necessary to consider also risk factors that can be identified and modified through specific lifestyle interventions.

Based on data demonstrating how lifestyle behaviors may be involved in both the genesis of severe psychiatric disorders and in increasing suicide risk (19), we aimed to provide a narrative review synthetizing existing published literature on lifestyle interventions and suicide prevention in psychiatric patients behavior. Additionally, we provide a review of the literature on lifestyle interventions promoting mental health reducing suicide risk in the general population and in patients with psychiatric disorders.

Lifestyle behaviors and interventions may differ at different life stages of life. Given this, we reviewed lifestyle behaviors and interventions associated with suicide risk in three age group categories: adolescents and young people (age 16–30), young adults and adults (age 30–65), and older adults and elderly (age >65).

## Materials and methods

We searched the relevant databases including MEDLINE, ISI Web of Knowledge – Web of Science Index, Cochrane Reviews Library and PsychoINFO for papers published from January 2000 to March 2018.

The following keywords were used: “lifestyle intervention,” “life satisfaction”, “diet,” “physical activity,” “nutrition,” “cholesterol,” “diabetes,” “lifestyle behaviors,” “cigarette smoke” matched with “suicide risk,” “healthy population,” “mental disorders,” “schizophrenia,” “psychotic disorders,” “bipolar disorder,” “depression,” “adolescents,” “adults,” and “elderly.” Inclusion criteria were: studies adopting psychosocial interventions for promoting changes in lifestyle behaviors, studies aiming to improve patients' physical status (evaluated in terms of body mass index – BMI, level of physical activity, smoking status, etc.) and studies that assess the association between lifestyle behaviors and psychiatric disorders and suicide risk. Only papers written in English were included. Studies included in this narrative review were those that examined suicide and lifestyle interventions in adolescence, young adults or adults and the elderly. Studies in which the age group investigated was not clearly defined were excluded from this review.

Randomized controlled trials, clinical controlled trials, pilot studies, cohort studies and reviews were considered eligible for the review process. In addition, the reference lists of all included studies and of relevant existing systematic reviews were checked for possible studies. Papers were screened by an independent researcher (IB) and assessed for eligibility by a senior expert (MP).

## Lifestyle behavior and suicide in adolescents and young people

Suicidal ideation and suicide attempt are relatively common in this phase of life; with completed suicide being the second most common cause of death in adolescents (20).

It has been described that 10–20% of adolescents suffer from a mental disorder (21). Adolescence is often associated with elevated levels of anxiety, stress, and adverse life events, which may lead to maladaptive feelings of hopelessness, personal failure, and suicidal ideation (22). While positive coping strategies, efficacious problem-solving skills, and general life satisfaction are considered protective for suicide (23). Common severe psychiatric disorders in adolescents include depression, anxiety disorders, behavioral problems (e.g., oppositional defiant disorder or conduct disorder), early psychosis, eating disorders (e.g., anorexia nervosa and bulimia nervosa) and addictive disorders (24), all risk factors for suicidal behavior in adolescents (25, 26).

Unhealthy lifestyle behaviors may impact mental health and suicidal behavior by influencing emotions and judgement. Studies have demonstrated that adolescents with first-episode psychosis have a high prevalence of tobacco, alcohol and cannabis use, selective dietary habits, lower physical activity, and lower level of activity during leisure time (27–30). Other studies have demonstrated a close relationship between cannabis use, hypomania, mania, and suicide risk in adolescents (31, 32). Furthermore, a relationship between weight, social relations, and depressive symptoms has been described (33); stressing the link between lifestyle behavior, mental health and suicide risk.

High symptom of any personality disorder in adolescence have negative repercussions on functioning over the subsequent 10 to 20 years. For instance, elevated Borderline Personality Disorder (BPD) symptoms in adolescence have been shown to be an independent risk factor for substance-use disorders during early adulthood, alcohol consumption, psychopathology, health risk behaviors, and suicide risk (34–37). Prevention and early intervention for BPD are necessary not only for reduction on psychiatric symptoms but also for improving psychosocial functioning and decreasing substance abuse, which are known risk factors for suicide.

Im et al. (38) examined 370,568 students with the aim of recognizing risk factors for suicidal ideation in adolescents found that low sleep satisfaction, high stress, alcohol consumption, smoking, and sexual activity, were significant lifestyle factors associated with suicidal ideation. Lee et al. (39) in a sample of 860 adolescents confirmed the relationship between several lifestyle behaviors and suicide risk including sleep disturbance, internet game addiction, and interpersonal factors (e.g., family conflicts and peer problems). A recent systematic review of 17 studies recognized that, in adolescents, a diagnosis of binge eating disorder was predictive of suicidality, while a high body mass index (BMI) was not (40). Substance-use disorders, including alcohol use disorder, are considered one of the most important risk factors associated with suicide risk during adolescence (41, 42). Moreover, it has been reported that the more the number of substances abused the higher the suicide risk (43). Borges et al. (44) in a recent trial on 1,071 young Mexicans reported that the use of cannabis before age 15, high frequency of cannabis use and another recent substance-use disorder increased the risk of suicide. Park et al. (45) collected data from 68,043 adolescents with the purpose of investigating the undesirable effects of frequent use of caffeinated energy drinks. Their results indicated that energy drink consumption was associated with sleep dissatisfaction, severe stress, depressive mood, suicidal ideation, suicide plan, and suicide attempt. Similarly, Kim et al. (46) in a study of 121,106 Korean adolescents confirmed that severe stress, inadequate sleep, and low school performance were associated with more energy drink consumption and suicide attempts.

Evidence suggests that sedentary lifestyle may impact emotional and mental health in adolescents (47). A systematic review on this topic found a strong relationship between sedentarism, depressive symptomatology, psychological distress, and suicide ideation in adolescents (48). In this regard, Lester et al. (49) demonstrated that participation in sports reduced the incidence of suicidal ideation.

Internet use has a mixed effect on children and young people's well-being. On one hand, internet users may develop a social support that may be artificial. On the other hand, it exposes adolescents to cyberbullying and meeting strangers online (50). In recent years, there has been an increased interest in the relationship between internet use, cyber-bullying, and suicide (51). A recent systematic review of 51 articles investigated the relationship between internet use and self-harm/suicidal behavior. This review demonstrated that internet addiction and elevated levels of internet use, were associated with a higher suicide risk (52). John et al. (53) highlighted that compared to non-victims of cyberbullying, victims of cyberbullying had a greater risk of both self-harm and suicidal behaviors. Rodelli et al. (54) evaluated the associations between physical activity, sport participation, healthy diet, higher sleep duration and lower levels of smoking and lower levels of alcohol use, and suicidal ideation when faced to cyberbullying. Results showed that cyberbullying victimization was associated with higher suicidal ideation while increased physical activity, sleeping longer, healthy diet and lower levels of smoking were associated with lower suicidal ideation. Moreover, a systematic review on the role of web technologies, mobile solutions, social networks, and machine learning in the prevention of suicide risk highlighted that many of these methods can help prevent suicide (55).

The potential role of family relationships (conflict, negative relationship with parents, other) as a risk factor for suicide was evaluated in 36,757 French adolescents (56). Results from this study demonstrated that negative relationships with parents and parental discord was significantly associated with suicide risk and/or depression in adolescents regardless of gender. The role of family interventions in the prevention of mental illness, substance abuse, and suicide ideation and attempts have been evaluated (57). The author suggested that family interventions could be useful in the prevention of suicidal ideation and behavior in both adolescents and parents.

## Lifestyle behavior and suicide in young adults and adults

In adults, the relationship between lifestyle behavior, severe psychiatric symptoms, and suicide risk is well documented (58, 59). Recent literature has demonstrated that behavioral lifestyle interventions help patients with serious mental illnesses lose weight and reduce cigarette smoking. Behavioral lifestyle interventions also helped patients achieve changes in fasting glucose levels in patients taking antipsychotic medications (60); simultaneously reducing the possibility of developing medical conditions; indirectly reducing the risk of suicide.

Studies have demonstrated that adult psychiatric inpatients who had attempted suicide presented higher levels of sedentarism and higher tobacco use compared to psychiatric patients who did not attempt suicide (61, 62).

Research reporting on the relationship between suicide, obesity, total serum cholesterol, and dietary patterns is controversial (63). Research examining the relationship between lipid profile and suicide attempts have shown that compared to non-attempters, suicide attempters had lower cholesterol levels (64, 65). More recently, a case control study of a Mexican population found a positive association between lower cholesterol levels and suicide attempt (66). Although the mechanism behind hypocholesterolemia and suicide has not been elucidated, it has been hypothesized that altered cholesterol at synaptic lipid rafts cause a decrease in serotonin transmission, neurotransmitter implicated in the pathophysiology of suicide (67, 68). A recent systematic review and meta-analysis of 11 studies evaluating lipid profile in suicide attempters and non-attempters in people with bipolar disorder failed to clarify the relationship between LDL-cholesterol, triglycerides and suicide in these patients. Shrivastava et al. (69) in a naturalistic cross-sectional cohort study of 60 patients with early psychosis, found that low levels of cholesterol were present only in the group of patients with severe suicidality. Similarly, Messaoud et al. (70) found a positive association between low plasma cholesterol level and suicidal behavior in patients with major depressive disorder.

Studies on the associations between depression, suicidal behavior, glucose levels or insulin resistance are scarce. Higher glucose levels have been associated with dysthymia (71) and higher HbA1c concentrations have been associated with psychotic depression (72). Koponen et al. (73) in 448 patients aged 35 years old found that patients with depression and suicidal behavior had higher blood glucose concentrations at baseline and at 2 h in the oral glucose tolerance test (OGTT).

Although dietary habits have been linked to depression, only few studies have examined the association between dietary patterns and suicide risk (74, 75). Li et al. (75) found that a dietary pattern comprise of vegetables, fruits, potatoes, soy products, mushrooms, seaweed and fish was associated with a decreased risk of suicide. Mukamal et al. (74) reviewed retrospectively the dietary information of 6,803 adults describing the differences in eating habits between suicide attempters and non-attempters. Results indicated that male attempters presented low consumption of vegetables and female attempters presented insufficient fruit consumption. It was further observed that female attempters ate significantly less fish and sea food compared to female non-attempters. Finally, results showed that fruits, vegetables, and meat were significantly under-consumed in those individuals with a history of suicide attempt.

The relationship between body mass index (BMI) and suicide risk in adult seems controversial. A study by Perera et al. (63) supported the hypothesis of an inverse association between BMI and completed suicide, where underweight was associated with an increased risk of completed suicide while obesity and overweight were associated with a decreased risk of suicide in comparison to people of normal weight. Contrarily, Branco et al. (76) stressed that obesity was associated with a higher prevalence of suicide risk, especially in women. Kim et al. (77) on 6,022 nationally representative sample of Korean adults aged 18 to 74 found that being underweight was associated with higher suicide risk and obesity was associated only with risk of suicide ideation but not with suicide attempt. In the same study, underweight individuals were more likely to report severe level of perceived stress (OR, 1.7; 95% Cl, 1.26–2.17) and life dissatisfaction (OR, 1.3; 95% Cl, 1.07–1.68) compared to obese individuals.

A strong relationship exists between well-being and physical activity, sports activities and level of fitness (78, 79). Physical activity and sports activities are considered protective lifestyle behaviuors against stress, depression and other unhealthy behaviors linked to medical illness. In particular, sport activities improve coronary functioning and reduce obesity prevalence (61, 80). Physical activity could be effective in reducing mental health disorders and suicidal behaviors through biological and psychosocial mechanisms; for example, releasing endorphins, increasing serotonin and norepinephrine synthesis, increasing the sense of mastery, self-esteem and social interaction (81). Vancampfort et al. (82) in a recent metanalysis showed that in adults, low physical activity was associated with higher suicide risk. Adams et al. (83) demonstrated that vigorous/moderate physical activity was associated with a positive perceived health and modestly associated with psychiatric symptoms and suicidal ideation reduction; confirming the link between physical activity and well-being.

In patients with schizophrenia, the evidence suggests that increasing physical exercise promotes wellbeing, and improves mental health in these patients (84). Dietary habits and levels of physical activity were evaluated in 428 people with schizophrenia spectrum disorders and increased waist circumference demonstrating that in these patients the intake of fiber, vegetables, fruit, and fish were insufficient and these patients also had low levels of physical activity. Furthermore, negative symptoms were associated with poor diet and less physical activity (85).

The positive relationship between smoking and suicide risk in adult patients with psychotic disorders has been evaluated in a systematic-review and a meta-analysis of observational studies on this topic (86). The same authors, in a randomized study, investigated the effect of smoking reduction on suicidality on 255 patients with a psychotic disorder. This author found that smoking reduction, besides the positive effects on physical health, had a protective effect on suicidal ideation in people with psychosis (87). Bhatt et al. (88) characterized and identified risk factors for suicide attempt in patients with psychiatric disorders. Findings from this study indicated no significant differences on demographic characteristics between psychiatric inpatients who attempted suicide and psychiatric inpatients who did not attempt suicide. The only risk factors for suicide in psychiatric patients found in this study were the presence of impulsivity and borderline personality symptoms. Howard et al. (89) in a recent population-based cohort study on 12,888 subjects (6,456 men, 6,432 women) investigated the relationship between chronic disease conditions, smoking habits, alcohol consumption, depressive symptoms, personality type, and other psychological parameters and suicide found that male sex, obesity, smoking, and living alone were associated with depression and with risk of suicide.

Several studies have focused on the role of occupation and work stressors on suicide risk. Research on this topic has shown that work characteristics and personal resources are linked to depression and suicide attempts (90). A study on 2,855 employees demonstrated that job autonomy, task variety, work-family conflict, family-work conflict, and job dissatisfaction contributed to suicide attempts (89). Furthermore, Kerr et al. (91) showed that poor interpersonal relationships, unemployment, debt and financial difficulties contribute to an increased risk of suicide in the general population. A recent study of 2,027 employed patients in primary care setting examined the association between workplace and suicidal ideation (92). Suicidality was significantly associated with work intensity in men and with work-related emotional demands in women.

Alcohol and substance abuse can worsen psychological well-being and contribute to suicide risk (93). Consumption of alcohol immediately prior to suicide is common (94, 95), with an estimated 37% of deaths from suicide having positive blood alcohol concentrations on toxicology screening indicating acute alcohol consumption before death (96). Bowden et al. (97) in a large cohort study of 2,803,457 residents of Wales, UK, highlighted the relationship between emergency alcohol-related hospital admission and the increased risk of suicide. A recent systematic review of 108 studies explored the associations between substance use and suicide risk in low- and middle-income countries (98) demonstrated an association between alcohol use, intoxication and pathological use of alcohol, tobacco, cannabis, illicit drugs and non-medical use of prescription drugs and suicide risk. Furthermore, Choi et al. (99) examined blood alcohol concentration (BAC) among suicide decedents aged 50 years or older. This study showed that alcohol problems prior to suicide, relationship problems and death/suicide of family/friends were associated with greater odds of having a positive BAC. This study also found that alcohol intoxication was linked to more violent means of suicide.

## Lifestyle behavior and suicide in older adults

Over 20% of adults aged 60 and over suffer from a psychiatric or neurological disorder. In the elderly, severe mental disorders present differently than in younger adults. The most common mental and neurological disorders in this age group are depression and dementia. However, anxiety disorders, schizophrenia and psychotic disorders, and substance use problems are also prevalent (100). A body of literature supports the association between late-life suicide ideation and various risk factors including depression and hopelessness (101, 102), while subjective well-being and meaning in life have been identified as protective factors for suicide in older adults (101). Innamorati et al. (103) in a study using psychological autopsy interviews studied 117 old-old adults who died by suicide and compared them to 97 young-old adults (6,574 years), 98 middle aged (50–64 years) suicide victims, and 117 psychiatric outpatients 75 years and older without a history of suicidal behaviors. They found that, in the elderly, unlike others stages of life, loneliness and lack of social support, were associated with risk of suicide. Physical illness, chronic severe pain, debilitating disease, and diagnosis of a terminal illness are common antecedents to suicide in older adults (104). Lee et al. (39) after analyzing age and sex-related features and suicide risk in the elderly stressed that the only stable risk factor for suicide in this age group was the negative perception of one's own health status.

Difficulties in interpersonal relations, social interactions, and social participation have been also linked to suicidal ideation and suicidal intent in the elderly (105). Mogensen et al. (106) reported that in the elderly, suicide risk appeared highest during the 6 months following the loss of a close relative and McLaren et al. (107) observed that widowhood was associated with suicidal ideation both in men and women. A longitudinal study on the importance of social support while adjusting to loneliness in bereaving elderly persons (108) and other studies examining on the importance of social supports reported that primary prevention programs designed specifically to increase social relations could decrease suicide risk (109). Older people are at risk for health decline and loss of independence that can affect social interactions negatively. Clark et al. (110) evaluated the effectiveness of lifestyle interventions in promoting well-being in independently living older people and Lapierre et al. (111) in a systematic review of 19 studies focusing on lifestyle programs for older adults, highlighted that psychoeducational programs and decreasing social isolation in this age group are effective interventions in the prevention of suicide. More recently, Okolie et al. (112) considered telephone counseling for vulnerable older adults and community-based programs incorporating education, gatekeeper training, depression screening, and group activities, as valid therapeutic options in preventing suicidal ideation. Lifestyle interventions in the areas of social interaction (113), personal goals (114), coping, and adaptive behavior (113), emotional flexibility (115), social skills (116), self-esteem (117), sense of belonging (118), reasons for living (119), hope (120), meaning in life (101, 121), religion or spirituality (122) could became promising directions to prevent suicide in older adults.

Several studies have demonstrated the importance of a balanced diet to prevent depression (123, 124) and of sleep-based interventions to prevent suicide (125). Considering that hypnotics use in the elderly has been associated with an increased risk of suicide (126), behavioral treatment of insomnia represents an efficacious alternative to pharmacotherapy in this age group (127). O' Rourke et al. (128) studied specific predictors of suicide in a sample of 220 older adults affected by Bipolar Disorder (BD). Alcohol misuse, medication non-adherence, and cognitive impairment were found to be direct predictors of suicide in these patients, while sleep disturbances acts as a risk factor on suicide ideation via depressive symptoms. Contrary to what happens in other age groups, a link between smoking prevalence and suicide rates in the elderly has not been observed (129).

## Discussion

In this article, we reviewed a growing body of literature demonstrating a relationship between lifestyle behaviors, mental health, and suicide risk (Table 1) in differing stages of life (Table 2) and in severe psychiatric disorders (Table 3). Our review suggests that enhancing protective factors and reducing risk factors known to increase suicidal behaviors (75, 130–132) could help prevent suicide in some individuals. Given the high comorbidity between psychiatric disorders and medical illnesses that result from unhealthy lifestyle behaviors, lifestyle interventions to reduce medical diseases, and increase patient's well-being are urgently needed.

**Table 1:** Lifestyle behaviors implicated in suicide risk (alphabetical order).

**Lifestyle behaviors**
•Difficulties in interpersonal relations
•Internet addiction
•Nutrition, dietary patterns
•Occupation and work stressor
•Sedentary
•Substance and alcohol abuse
•Tobacco's smoke
•Underweight, Obesity

**Table 2:** Common Lifestyle behaviors implicated in suicide risk in different age ranges (based on results of studies included in this review).

**Age range**	**Lifestyle behavior and suicide risk**
Adolescents and young people (age 16–30)	Difficulties in interpersonal relations, substance and alcohol abuse, internet addiction.
Young adults and adults (age 30–65)	Substance and alcohol abuse, occupation and work stressor.
Older adults and elderly (age >65)	Difficulties in interpersonal relations, nutrition, dietary patterns.

**Table 3:** Common Lifestyle behaviors implicated in suicide risk in psychiatric disorders (based on results of studies included in this review).

**Psychiatric disorders**	**Lifestyle behavior and suicide risk**
Psychotic disorders	Substance and alcohol abuse, difficulties in interpersonal relations, tobacco's smoke.
Depressive disorders	Substance and alcohol abuse, occupation and work stressor.

The relationship between lifestyle behavior and suicide is complex and multifactorial. Unhealthy lifestyle behaviors can directly increase the risk of suicide; at the same time increasing the risk for many medical diseases. It is well known that medical illnesses are associated with disability, social isolation and associated with an increased risk of suicide. Sedentary behaviors, underweight, obesity, cigarette smoking, and alcohol abuse are associated with cardiometabolic risk factors, poor mental health, and severe psychiatric disorders. These factors contribute to an increased risk of suicide. Sedentary behaviors, weight issues and fewer social supports contribute to social isolation, limiting the development of social relationships, which increase the risk of developing mental health problems and suicide. Adopting healthy lifestyle behaviors are indispensable for both, improving health and for the prevention of suicide at any stage in life.

Studies included in this article showed different lifestyle behaviors in different lifestages. In adolescent and young adults there are multiple important risk factors for suicide including psychosis, depression, low sleep satisfaction, high stress, substance abuse, alcohol consumption, smoking, sexual activity, internet addiction, interpersonal factors, personality disorders, anxiety or conduct disorders, eating disorders, and aggressive, irritable and antisocial tendencies (38, 39). Though it has been demonstrated that school-based suicide prevention programs increase knowledge on suicide without really preventing suicidal behaviors (133), it seems necessary to consider prevention programs that identify substance abuse, educational competencies (literacy, study skills, time management), educational environment, school programs, social interactions, academic attainment, cognitive progress, emotional control, behavioral expectations, physical and moral development, and encouragement of active engagement in sports.

In adults, lifestyle interventions on smoking, heavy alcohol consumption, physical inactivity, BMI, sedentarism, and eating habits should play a significant role in suicide prevention (17, 65). Effective lifestyle prevention programs in adults can be developed through screening programs identifying and assessing at-risk groups. These programs should include lifestyle training, psychoeducation, support/skills training and crisis response and referral resources (134). In patients with severe psychiatric disease, strategies that promote exercise and sport activities, that reduce caffeine consumption and other health-adverse behaviors are particularly important for the reduction of psychotic symptoms, depression, anxiety, low self-esteem, and life dissatisfaction, factors that increase suicide risk. Gallego et al. (135) suggested that in patients with either schizophrenia spectrum disorders or affective disorders, physicians should be alert for the presence of a previous suicidal attempts and financial or relationship losses, factors that can increase suicidal risk. Furthermore, psychiatric hospitalization, a close psychiatric follow up, collaboration with family would be important strategies in reducing the risk of suicidal behavior in patients affected by severe psychiatric disorders.

Finally, in the elderly, difficulties in interpersonal relations, social isolation and the consequences of physical disability can represent targeted and specific points for intervention. The concomitant presence of depressive symptoms and stressing life events including loneliness and physical illness should be considered warning signs for suicidal risk (136). In the elderly, innovative strategies should promote positive aging, involve family and community gatekeepers, and use telecommunications to identify older adult at risk of suicide (112). Psychosocial interventions influence depressive symptoms and considering that mental illness is one of the most significant factors in suicide; psychosocial interventions should be part of suicide prevention programs in the elderly. Further trials are warranted, especially for the most promising type of interventions for preventing suicide, that is, social activities (137, 138).

## Limitations

When interpreting the results of this review, several limitations should be considered. First, this is a narrative review, therefore, studies were subjectively selected, which could have led to selection bias. Second, we only selected articles in English leaving relevant articles in other languages out of our review. Finally, we did not draw a distinction between gender and risk factors for suicide.

## Conclusions

Lifestyle behavior is of paramount importance for suicide prevention as maladaptive maneuvers (such as drinking or drug abuse) as to face increased psychological pain and distress consistently reduce cognitive skill to solve source of suffering. Furthermore, reduced quality of life as observed by impairment of some psychiatric disorders can be worsened by low exercise, excessive smoking, poor diet, and determinants associated with pharmacological treatment. Community mental health with proper programs such as assertive community treatment, psychoeducational family treatment, social skills training, and psychosocial therapies are important interventions that can incorporate education on lifestyle and ultimately reduce suicide risk.

## Author contributions

IB and VC searched the literature and provided first draft. MH, AC, and DE independently reviewed the paper and added relevant information. MP provided the intellectual impetuous and supervised the entire process of development of the paper.

### Conflict of interest statement

The authors declare that the research was conducted in the absence of any commercial or financial relationships that could be construed as a potential conflict of interest.

## References

[1] Laursen TM Munk-Olsen T Vestergaard M. Life expectancy and cardiovascularmortality in persons with schizophrenia. Curr Opin Psychiatry (2012) 25:83–8. 10.1097/YCO.0b013e32835035ca 22249081

[2] Laursen TM Nordentoft M Mortensen PB. Excess early mortality in schizophrenia. Annu Rev Clin Psychol. (2013) 10:425–48. 10.1146/annurev-clinpsy-032813-153657 24313570

[3] De Hert M Correll CU Bobes J Cetkovich-Bakmas M Cohen D Asai I Physical illness in patients with severe mental disorders. I. Prevalence, impact of medications and disparities in health care. World Psychiatry (2011) 10:52–77. 10.1002/j.2051-5545.2011.tb00014.x 21379357 PMC3048500

[4] Smith DJ Langan J McLean G Guthrie B Mercer SW. Schizophrenia is associated with excess multiple physical-health comorbidities but low levels of recorded cardiovascular disease in primary care: cross-sectional study. BMJ Open (2013) 3:e002808. 10.1136/bmjopen-2013-002808 23599376 PMC3641427

[5] Zhang J Xiao S Zhou L. Mental disorders and suicide among young rural Chinese: a case-control psychological autopsy study. Am J Psychiatry (2010) 167:773–81. 10.1176/appi.ajp.2010.09101476 20395398 PMC3210861

[6] Tousignant M Pouliot L Routhier D Vrakas G McGirr A Turecki G. Suicide,schizophrenia, and schizoid-type psychosis: role of life events and childhoodfactors. Suicide Life Threat Behav. (2011) 41:66–78. 10.1111/j.1943-278X.2010.00002.x 21309825

[7] Park S Lee Y Youn T Kim BS Park JI Kim H. Association between level of suicide risk, characteristics of suicide attempts, and mental disorders among suicide attempters. BMC Public Health (2018) 18:477. 10.1186/s12889-018-5387-8 29642887 PMC5896087

[8] Bruins J Jörg F Bruggeman R Slooff C Corpeleijn E Pijnenborg M. The effects of lifestyle interventions on (long-term) weight management, cardiometabolic risk and depressive symptoms in people with psychotic disorders: a meta-analysis. PLoS ONE (2014) 9:e112276. 10.1371/journal.pone.0112276 25474313 PMC4256304

[9] Sarris J O'Neil A Coulson CE Schweitzer I Berk M. Lifestyle medicine for depression. BMC Psychiatry (2014) 14:107. 10.1186/1471-244X-14-107 24721040 PMC3998225

[10] Sharifi V Eaton WW Wu LT Roth KB Burchett BM Mojtabai R. Psychoticexperiences and risk of death in the general population: 24-27 year follow-up of the Epidemiologic Catchment Area study. Br J Psychiatry (2015) 207:30–6. 10.1192/bjp.bp.113.143198 25953893 PMC4486819

[11] Bray I Gunnell D. Suicide rates, life satisfaction and happiness as markers for population mental health. Soc Psychiatry Psychiatr Epidemiol. (2006) 41:333–7. 10.1007/s00127-006-0049-z 16565916

[12] Shmotkin D Happiness in the face of adversity: reformulating the dynamic and modular bases of subjective well-being. Rev Gen Psych. (2005) 9:291–325. 10.1037/1089-2680.9.4.291

[13] Shrira A Palgi Y Ben-Ezra M Shmotkin D How subjective well-being and meaning in life interact in the hostile world? J Pos Psych. (2011) 6:273–85. 10.1080/17439760.2011.577090

[14] Prendergast KB Schofield GM Mackay LM. Associations between lifestyle behaviours and optimal wellbeing in a diverse sample of New Zealand adults. BMC Public Health (2016) 16:62. 10.1186/s12889-016-2755-0 26801097 PMC4722793

[15] Chiu HF Dai J Xiang YT Chan SS Leung T Yu X Suicidal thoughts and behaviours in older adults in rural China: a preliminary study. Int J Geriatr Psychiatry (2012) 27:1124–30. 10.1002/gps.2831 22252964 PMC4733507

[16] Voracek M. National intelligence, suicide rate, and subjective well-being. Percept Mot Skills (2009) 109:718–20. 10.2466/pms.109.3.718-720 20178270

[17] Koivumaa-Honkanen H Honkanen R Koskenvuo M Kaprio J. Self-reported happiness in life and suicide in ensuing 20 years. Soc Psychiatry Psychiatr Epidemiol. (2003) 38:244–8. 10.1007/s00127-003-0625-4 12719839

[18] Mercy JA Rosenberg ML. Building a foundation for suicide prevention: the contributions of Jack, C. Smith Am J Prev Med. (2000) 19:26–30. 10.1016/S0749-3797(00)00170-7 10863127

[19] De Rosa C Sampogna G Luciano M Del Vecchio V Pocai B Borriello G Improving physical health of patients with severe mental disorders: a critical review of lifestyle psychosocial interventions. Expert Rev Neurother. (2017) 17:667–81. 10.1080/14737175.2017.1325321 28468528

[20] Lunde I Myhre Reigstad M Frisch Moe K Grimholt TK. Systematic Literature Review of Attempted Suicide and Offspring. Int J Environ Res Public Health. (2018) 15:E937. 10.3390/ijerph15050937 29738447 PMC5981976

[21] Brooks TL Harris SK Thrall JS Woods ER Association of adolescent risk behaviours with mental health symptoms in high school students. J Adolesc Health (2002) 31:240–6. 10.1016/S1054-139X(02)00385-3 12225736

[22] Serafini G Muzio C Piccinini G Flouri E Ferrigno G Pompili M Life adversities and suicidal behaviour in young individuals: a systematic review. Eur Child Adolesc Psychiatry (2015) 24:1423–46. 10.1007/s00787-015-0760-y 26303813

[23] Montross LP Zisook S Kasckow J. Suicide among patients with schizophrenia: a consideration of risk and protective factors. Ann Clin Psychiatry (2005) 17:173–82. 10.1080/10401230591002156 16433060

[24] Kessler RC Berglund P Demler O Jin R Merikangas KR Walters EE. Lifetime prevalence and age-of-onset distributions of DSM-IV disorders in the National Comorbidity Survey replication. Arch Gen Psychiatry (2005) 62:593–602. 10.1001/archpsyc.62.6.593 15939837

[25] McGinty J Sayeed Haque M Upthegrove R. Depression during first episode psychosis and subsequent suicide risk: a systematic review and meta-analysis of longitudinal studies. Schizophr Res. (2017) 195:58–66. 10.1016/j.schres.2017.09.040 28982553

[26] Orri M Galera C Turecki G Forte A Renaud J Boivin M. Association of childhood irritability and depressive/anxious mood profiles with adolescent suicidal ideation and attempts. JAMA Psychiatry (2018) 75:465–73. 10.1001/jamapsychiatry.2018.0174 29590281 PMC5875380

[27] Myles N Newall HD Curtis J Nielssen O Shiers D Large M. Tobacco use before, at, and after first-episode psychosis: a systematic meta-analysis. J Clin Psychiatry (2012) 73:468–75. 10.4088/JCP.11r07222 22579146

[28] Pawełczyk T Trafalska E Pawełczyk A Kotlicka-Antczak M. Differences in omega-3 and omega-6 polyunsaturated fatty acid consumption in people at ultra-high risk of psychosis, first-episode schizophrenia, and in healthy controls. Early Interv Psychiatry (2017) 11:498–508. 10.1111/eip.12267 26279283

[29] Sormunen E Saarinen MM Salokangas RKR Telama R Hutri-Kähönen N Tammelin T. Effects of childhood and adolescence physical activity patterns on psychosis risk-a general population cohort study. NPJ Schizophr. (2017) 3:5. 10.1038/s41537-016-0007-z 28560251 PMC5441534

[30] Oluwoye O Monroe-DeVita M Burduli E Chwastiak L McPherson S McClellan JM. Impact of tobacco, alcohol and cannabis use on treatment outcomes among patients experiencing first episode psychosis: data from the national RAISE-ETP study. Early Interv Psychiatry (2018) [Epub ahead of print]. 10.1111/eip.12542 29356438 PMC6684200

[31] Leite RT Nogueira Sde O do Nascimento JP de Lima LS da Nóbrega TB Virgínio Mda S. The use of cannabis as a predictor of early onset of bipolar disorder and suicide attempts. Neural Plast. (2015) 2015:434127. 10.1155/2015/434127 26097750 PMC4444580

[32] Marwaha S Winsper C Bebbington P Smith D. Cannabis use and hypomania in young people: a prospective analysis. Schizophr Bull. 44:1267–74. 10.1093/schbul/sbx158 29207008 PMC6192498

[33] Brewis AA Bruening M. Weight Shame, Social connection, and depressive symptoms in late adolescence. Int J Environ Res Public Health (2018) 15:E891. 10.3390/ijerph15050891 29723962 PMC5981930

[34] Chanen A. M. McCutcheon L. (2013). Prevention and early intervention for borderline personality disorder: current status and recent evidence. Br J Psychiatry 54(Suppl.):s24–9. 10.1192/bjp.bp.112.119180 23288497

[35] Cohen P Crawford TN Johnson JG Kasen S. The children in the community study of developmental course of personality disorder. J Pers Disord. (2005) 19:466–86. 10.1521/pedi.2005.19.5.466 16274277

[36] Winograd G Cohen P Chen H. Adolescent borderline symptoms in the community: prognosis for functioning over 20 years. J Child Psychol Psychiatry (2008) 49:933–41. 10.1111/j.1469-7610.2008.01930.x 18665882

[37] Oldham JM. Borderline personality disorder and suicidality. Am J Psychiatry (2006) 163, 20–6. 10.1176/appi.ajp.163.1.20 16390884

[38] Im Y Oh WO Suk M. Risk factors for suicide ideation among adolescents: five-year national data analysis. Arch Psychiatr Nurs. (2017) 31:282–6. 10.1016/j.apnu.2017.01.001 28499568

[39] Lee H Seol KH Kim JW. Age and sex-related differences in risk factors for elderly suicide: Differentiating between suicide ideation and attempts. Int J Geriatr Psychiatry (2018) 33:e300–e306. 10.1002/gps.4794 28967671

[40] Conti C Lanzara R Scipioni M Iasenza M Guagnano MT Fulcheri M. The relationship between binge eating disorder and suicidality: a systematic review. Front Psychol. (2017) 8:2125. 10.3389/fpsyg.2017.02125 29259574 PMC5723427

[41] Galaif ER Sussman S Newcomb MD Locke TF. Suicidality, depression, and alcohol use among adolescents: a review of empirical findings. Int J Adolesc Med Health (2007) 19:27–35. 10.1515/IJAMH.2007.19.1.27 17458321 PMC3134404

[42] Pompili M Serafini G Innamorati M Biondi M Siracusano A Di Giannantonio M Substance abuse and suicide risk among adolescents. Eur Arch Psychiatry Clin Neurosci. (2012) 262:469–85. 10.1007/s00406-012-0292-0 22290639

[43] Kokkevi A Richardson C Olszewski D Matias J Monshouwer K Bjarnason T. Multiple substance use and self-reported suicide attempts by adolescentsin 16 European countries. Eur Child Adolesc Psychiatry (2012) 21:443–50. 10.1007/s00787-012-0276-7 22535305

[44] Borges G Benjet C Orozco R Medina-Mora ME Menendez D. Alcohol, cannabis and other drugs and subsequent suicide ideation and attempt among young Mexicans. J Psychiatr Res. (2017) 91:74–82. 10.1016/j.jpsychires.2017.02.025 28325681

[45] Park S Lee Y Lee JH. Association between energy drink intake, sleep, stress, and suicidality in Korean adolescents: energy drink use in isolation or in combination with junk food consumption. Nutr J. (2016). 15:87. 10.1186/s12937-016-0204-7 27737671 PMC5064784

[46] Kim SY Sim S Choi HG. High stress, lack of sleep, low school performance, and suicide attempts are associated with high energy drink intake in adolescents. PLoS ONE (2017) 12:e0187759. 10.1371/journal.pone.0187759 29135989 PMC5685612

[47] Teychenne M Costigan SA Parker K. The association between sedentarybehaviour and risk of anxiety: a systematic review. BMC Public Health (2015) 15:513. 10.1186/s12889-015-1843-x 26088005 PMC4474345

[48] Hoare E Milton K Foster C Allender S. The associations between sedentary behaviour and mental health among adolescents: a systematic review. Int J Behav Nutr Phyc Act. (2016) 13:108. 10.1186/s12966-016-0432-4 27717387 PMC5055671

[49] Lester D Participation in sports teams and suicidal behaviour: an analysis of the 1995 national college health risk behaviour survey. Percept Mot Skills (2014) 119:38–41. 10.2466/06.15.PMS.119c13z5 25153736

[50] Robertson L Skegg K Poore M Williams S Taylor B. An adolescent suicide cluster and the possible role of electronic communication technology. Crisis (2012) 33:239–45. 10.1027/0227-5910/a000140 22562859

[51] Messina ES Iwasaki Y Internet use and self-injurious behaviours among adolescents and young adults: an interdisciplinary literature review and implications for health professionals. Cyberpsychol Behav Soc Netw. (2011) 14:161–8. 10.1089/cyber.2010.0025 20677983

[52] Marchant A Hawton K Stewart A Montgomery P Singaravelu V Lloyd K. A systematic review of the relationship between internet use, self-harm and suicidal behaviour in young people: the good, the bad and the unknown. PLoS ONE (2017) 12:e0181722. 10.1371/journal.pone.0181722 28813437 PMC5558917

[53] John A Glendenning AC Marchant A Montgomery P Stewart A Wood S. Self-Harm, suicidal behaviours, and cyberbullying in children and young people: systematic review. J Med Internet Res. (2018) 20:e129. 10.2196/jmir.9044 29674305 PMC5934539

[54] Rodelli M De Bourdeaudhuij I Dumon E Portzky G DeSmet A. Which healthy lifestyle factors are associated with a lower risk of suicidal ideation among adolescents faced with cyberbullying? Prev Med. (2018) 113:32–40. 10.1016/j.ypmed.2018.05.002 29729287

[55] Franco-Martín M. A. Muñoz-Sánchez J. L. Sainz-de-Abajo B. Castillo-Sánchez G. Hamrioui S. de la Torre-Díez I. (2018). A systematic literature review of technologies for suicidal behaviour prevention. J Med Syst. 42:71 10.1007/s10916-018-0926-5 29508152

[56] Consoli A Peyre H Speranza M Hassler C Falissard B Touchette E Suicidal behaviours in depressed adolescents: role of perceived relationships in the family. Child Adolesc Psychiatry Ment Health (2003) 7:8 10.1186/1753-2000-7-8 PMC3655930 23497551

[57] Brent D. Prevention Programs to Augment Family and Child Resilience Can Have Lasting Effects on Suicidal Risk. Suicide Life Threat Behav. (2016) 46(Suppl. 1):S39–47. 10.1111/sltb.12257 27094110

[58] Diaz FJ James D Botts S Maw L Susce MT de Leon J Tobacco smoking behaviours in bipolar disorder: a comparison of the general population, schizophrenia, and major depression. Bipolar Disord. (2009) 11:154–65. 10.1111/j.1399-5618.2009.00664.x 19267698

[59] Bostock EC Kirkby KC Taylor BV. The Current Status of the Ketogenic Diet in Psychiatry. Front Psychiatry (2017) 8:43. 10.3389/fpsyt.2017.00043 28373848 PMC5357645

[60] Green CA Yarborough BJ Leo MC Yarborough MT Stumbo SP Janoff SL. The STRIDE weight loss and lifestyle intervention for individuals taking antipsychotic medications: a randomized trial. Am J Psychiatry (2015) 172:71–81. 10.1176/appi.ajp.2014.14020173 25219423 PMC4282602

[61] Andriessen K Krysinska K Can sport events affect suicidal behaviour? A review of the literature and implications for prevention. Crisis (2009) 30:144–52. 10.1027/0227-5910.30.3.144 19767270

[62] Poorolajal J. Darvishi N. (2016). Smoking and suicide: a meta-analysis. PLoS ONE 11:e0156348 10.1371/journal.pone.0156348 27391330 PMC4938402

[63] Perera S Eisen RB Dennis BB Bawor M Bhatt M Bhatnagar N. Body mass index is an important predictor for suicide: results from a systematic review and meta-analysis. Suicide Life Threat Behav. (2016) 46:697–736. 10.1111/sltb.12244 27094229

[64] Diaz-Sastre C Baca-Garcia E Perez-Rodriguez MM Garcia-Resa E Ceverino A Saiz-Ruiz J. Low plasma cholesterol levels in suicidal males: a gender- and body mass index-matched case-control study of suicide attempters and nonattempters. Prog Neuropsychopharmacol Biol Psychiatry (2007) 31:901–5. 10.1016/j.pnpbp.2007.02.004 17363125

[65] Wu S Ding Y Wu F Xie G Hou J Mao P. Serum lipid levels and suicidality: a meta-analysis of 65 epidemiological studies. J Psychiatry Neurosci. (2016) 41:56–69. 10.1503/jpn.150079 26505144 PMC4688029

[66] Segoviano-Mendoza M Cárdenas-de la Cruz M Salas-Pacheco J Vázquez-Alaniz F La Llave-León O Castellanos-Juárez F. Hypocholesterolemia is an independent risk factor for depression disorder and suicide attempt in Northern Mexican population. BMC Psychiatry (2018) 18:7. 10.1186/s12888-018-1596-z 29334911 PMC5769344

[67] Bartoli F Di Brita C Crocamo C Clerici M Carrà G. Lipid profile and suicide attempt in bipolar disorder: A meta-analysis of published and unpublished data. Prog Neuropsychopharmacol Biol Psychiatry (2017) 79 (Pt B):90–5. 10.1016/j.pnpbp.2017.06.008 28627446

[68] Mathew B Srinivasan K Pradeep J Thomas T Mandal AK. Suicidal behaviour is associated with decreased esterified cholesterol in plasma and membrane fluidity of platelets. Asian J Psychiatr. (2018) 32:105–9. 10.1016/j.ajp.2017.11.023 29222984

[69] Shrivastava A Johnston M Campbell R De Sousa A Shah N Serum cholesterol and Suicide in first episode psychosis: a preliminary study. Indian J Psychiatry (2017) 5:478–82. 10.4103/psychiatry.IndianJPsychiatry_185_17 PMC5806328 29497191

[70] Messaoud A Mensi R Mrad A Mhalla A Azizi I Amemou B. Is low total cholesterol levels associated with suicide attempt in depressive patients? Ann Gen Psychiatry (2017) 16:20. 10.1186/s12991-017-0144-4 28428806 PMC5392998

[71] Ceretta LB Réus GZ Abelaira HM Jornada LK Schwalm MT Hoepers NJ. Increased prevalence of mood disorders and suicidal ideation in type 2 diabetic patients. Acta Diabetol. (2012) 49(Suppl. 1):S227–34. 10.1007/s00592-012-0435-9 23064949

[72] Han SJ Kim HJ Choi YJ Lee KW Kim DJ. Increased risk of suicidal ideation in Korean adults with both diabetes and depression. Diabetes Res Clin Pract. (2013) 101:e14–7. 10.1016/j.diabres.2013.06.012 23871574

[73] Koponen H Kautiainen H Leppänen E Mäntyselkä P Vanhala M. Association between suicidal behaviour and impaired glucose metabolism in depressive disorders. BMC Psychiatry (2015) 15:163. 10.1186/s12888-015-0567-x 26199013 PMC4509469

[74] Mukamal KJ Kawachi I Miller M Rimm EB. Body mass index and risk of suicide among men. Arch Intern Med. (2007) 167:468–75. 10.1001/archinte.167.5.468 17353494

[75] Li Y Zhang J McKeown RE. Cross-sectional assessment of diet quality in individuals with a lifetime history of attempted suicide. Psychiatry Res. (2009) 165:111–9. 10.1016/j.psychres.2007.09.004 19046606

[76] Branco JC Motta J Wiener C Oses JP Pedrotti Moreira F Spessato B Association between obesity and suicide in woman, but not in man: a population-based study of young adults. Psychol Health Med. (2017) 2:275–81. 10.1080/13548506.2016.1164870 27006170

[77] Kim H Jeon HJ Bae JN Cho MJ Cho SJ Lee H Association of body mass index with suicide behaviours, perceived stress, and life dissatisfaction in the Korean general population. Psychiatry Investig. (2018) 15:272–8. 10.30773/pi.2017.06.28 PMC5900366 29486542

[78] Pate RR Trost SG Levin S Dowda M Sports participation and health-related behaviours among US youth. Arch Pediatr Adolesc Med. (2000) 154:904–11. 10.1001/archpedi.154.9.904 10980794

[79] Warburton DER Nicol CW Bredin SSD. Health benefits of physical activity: The evidence. Can Med Assoc, J. (2006) 174:801–9. 10.1503/cmaj.051351 16534088 PMC1402378

[80] Babiss LA Gangwisch JE. Sports participation as a protective factor against depression and suicidal ideation in adolescents as mediated by self-esteem and social support. J Dev Behav Pediatr. (2009) 30:376–84. 10.1097/DBP.0b013e3181b33659 19692930

[81] Brosnahan J Steffen LM Lytle L Patterson J Boostrom A. The relation between physical activity and mental health among Hispanic and non-Hispanic white adolescents. Arch Pediatr Adolesc Med. (2004) 158:818–23. 10.1001/archpedi.158.8.818 15289257

[82] Vancampfort D Hallgren M Firth J Rosenbaum S Schuch FB Mugisha J. Physical activity and suicidal ideation: A systematic review and meta-analysis. J Affect Disord. (2018) 225:438–48. 10.1016/j.jad.2017.08.070 28858658

[83] Adams TB Moore MT Dye J. The relationship between physical activity and mental health in a national sample of college females. Women Health (2007) 45:69–85. 10.1300/J013v45n01_05 17613463

[84] Bassilios B Judd F Pattison P Nicholas A Moeller-Saxone K Predictors of exercise in individuals with schizophrenia, A test of the transtheoretical model of behaviour change. Clin Schizophr Relat Psychoses (2015) 8:173–82. 10.3371/CSRP.BAJU.030113 23471086

[85] Jakobsen AS Speyer H Nørgaard HCB Karlsen M Hjorthøj C Krogh J. Dietary patterns and physical activity in people with schizophrenia and increased waist circumference. Schizophr Res. (2018) S0920-9964(18)30168-3. 10.1016/j.schres.2018.03.016 29555213

[86] Sankaranarayanan A Mancuso S Wilding H Ghuloum S Castle D. Correction: smoking, suicidality and psychosis: a systematic meta-analysis. PLoS ONE (2015) 10:e0141024. 10.1371/journal.pone.0141024 26372218 PMC4570823

[87] Sankaranarayanan A Clark V Baker A Palazzi K Lewin TJ Richmond R. Reducing smoking reducessuicidality among individuals with psychosis: Complementary outcomes from aHealthy Lifestyles intervention study. Psychiatry Res. (2016) 243:407–12. 10.1016/j.psychres.2016.07.006 27450743

[88] Bhatt M Perera S Zielinski L Eisen RB Yeung S El-Sheikh W. Profile of suicide attempts and risk factors among psychiatric patients: a case-control study. PLoS ONE (2018) 13:e0192998. 10.1371/journal.pone.0192998 29470514 PMC5823369

[89] Howard M Krannitz M. A Reanalysis of Occupation and Suicide: Negative Perceptions of the Workplace Linked to Suicide Attempts. J Psychol. (2017) 151:767–88. 10.1080/00223980.2017.1393378 29166224

[90] Hsu CY Chang SS Yip PSF Subjective wellbeing, suicide and socioeconomic factors: an ecological analysis in Hong Kong. Epidemiol Psychiatr Sci. (2018) 10:1–19. 10.1017/S2045796018000124 PMC6998950 29633681

[91] Kerr WC Kaplan MS Huguet N Caetano R Giesbrecht N McFarland BH. Economic recession, alcohol, and suicide rates: comparative effects of poverty, foreclosure, and job loss. Am J Prev Med. (2017) 52:469–75. 10.1016/j.amepre.2016.09.021 27856114 PMC5362282

[92] Younès N Rivière M Plancke L Leroyer A Blanchon T Da Silva MA. Work intensity in men and work-related emotional demands in women are associated with increased suicidality among persons attending primary care. J Affect Disord. (2018) 235:565–73. 10.1016/j.jad.2018.04.075 29698918

[93] Darvishi N Farhadi M Haghtalab T Poorolaja J. Alcohol-related risk of suicidal ideation, suicide attempt, and completed suicide: a meta-analysis. PLoS ONE (2015) 10:e0126870. 10.1371/journal.pone.0126870 25993344 PMC4439031

[94] Bedford D O'Farrell A Howell F. Blood alcohol levels in persons who died from accidents and suicide. Ir Med J. (2006) 99:80–3. 16700260

[95] Kaplan MS Huguet N McFarland BH Caetano R Conner KR Giesbrecht N. Use of alcohol before suicide in the United States. Ann Epidemiol. (2014) 24:588–592.e1-2. 10.1016/j.annepidem.2014.05.008 24953567 PMC4119510

[96] Cherpitel CJ Borges GL Wilcox HC Acute alcohol use and suicidal behaviour: a review of the literature. Alcohol Clin Exp Res. (2004) 28(5 Suppl):18S−28S. 10.1097/01.ALC.0000127411.61634.14 15166633

[97] Bowden B John A Trefan L Morgan J Farewell D Fone D. Risk of suicide following an alcohol-related emergency hospital admission: an electronic cohort study of 2.8 million people. PLoS ONE (2018) 13:e0194772. 10.1371/journal.pone.0194772 29702655 PMC5922531

[98] Breet E Goldstone D Bantjes J. Substance use and suicidal ideation and behaviour in low- and middle-income countries: a systematic review. BMC Public Health (2018) 18:549. 10.1186/s12889-018-5425-6 29699529 PMC5921303

[99] Choi NG DiNitto DM Sagna AO Marti CN. Postmortem blood alcohol content among late-middle aged and older suicide decedents: associations with suicide precipitating/risk factors, means, and other drug toxicology. Drug Alcohol Depend. (2018) 187:311–8. 10.1016/j.drugalcdep.2018.02.034 29704853

[100] Almeida OP Hankey GJ Yeap BB Golledge J Norman PE Flicker L. Mortality among people with severe mental disorders who reach old age: a longitudinal study of a community-representative sample of 37892 men. PLoS ONE (2014) 9:e111882. 10.1371/journal.pone.0111882 25360781 PMC4216120

[101] Heisel MJ Flett GL. Does recognition of meaning in life confer resiliency to suicide ideation among community-residing older adults? A longitudinal investigation. Am J Geriatr Psychiatry (2016) 24:455–66. 10.1016/j.jagp.2015.08.007 26880611

[102] Szanto K Galfalvy H Vanyukov PM Keilp JG Dombrovski AY Pathways to late-life suicidal behaviour: cluster analysis and predictive validation of suicidal behaviour in a sample of older adults with major depression. J Clin Psychiatry (2018) 79:11611 10.4088/JCP.17m11611 PMC5932247 29489076

[103] Innamorati M Pompili M Di Vittorio C Baratta S Masotti V Badaracco A. Suicide in the old elderly: results from one Italian county. Am J Geriatr Psychiatry (2014) 22:1158–67. 10.1016/j.jagp.2013.03.003 23890752

[104] Lutz J Fiske A Functional disability and suicidal behaviour in middle-aged and older adults: a systematic critical review. J Affect Disord. (2018) 227:260–71. 10.1016/j.jad.2017.10.043 29107819

[105] Fässberg MM van Orden KA Duberstein P Erlangsen A Lapierre S Bodner E A systematic review of social factors and suicidal behaviour in older adulthood. Int J Environ Res Public Health (2012) 9:722–45. 10.3390/ijerph9030722 22690159 PMC3367273

[106] Mogensen H Moller J Hultin H and Mittendorfer-Rutz E. Death of a close relative and the risk of suicide in Sweden-A Large Scale Register-Based Case-Crossover Study. PLoS ONE (2016) 11:e0164274. 10.1371/journal.pone.0164274 27727324 PMC5058490

[107] McLaren S Gomez R Gill P Chesler J. Marital status and suicidal ideation among Australian older adults: the mediating role of sense of belonging. Int Psychogeriatr. (2015) 27:145–54. 10.1017/S1041610214001501 25101552

[108] van Baarsen B Theory of social support and self-esteem on adjustment to emotional and social loneliness following a partner's death in later life. J Gerontol B Psychol Sci Soc Sci. (2002) 57:S33–42. 11773231 10.1093/geronb/57.1.s33

[109] Erlangsen A Nordentoft M, Conwell Y, Waern M, De Leo D, Lindner R, et al. Key considerations for preventing suicide in older adults: consensus opinions of an expert panel. Crisis (2001) 32:106–9. 10.1027/0227-5910/a000053 21616757

[110] Clark F Jackson J Carlson M Chou CP Cherry BJ Jordan-Marsh M. Effectiveness of a lifestyle intervention in promoting the well-being of independently living older people: results of the Well Elderly 2 Randomised Controlled Trial. J Epidemiol Community Health. (2012) 66:782–90. 10.1136/jech.2009.099754 21636614 PMC3412049

[111] Lapierre S Erlangsen A Waern M De Leo D Oyama H Scocco P. A systematic review of elderly suicide prevention programs. Crisis (2011) 32:88–98. 10.1027/0227-5910/a000076 21602163 PMC3728773

[112] Okolie C Dennis M Simon Thomas E John A A systematic review of interventions to prevent suicidal behaviours and reduce suicidal ideation in older people. Int Psychogeriatr. (2017) 29:1801–24. 10.1017/S1041610217001430 28766474

[113] Heisel MJ Duberstein PR Talbot NL King DA Tu XM. Adapting Interpersonal psychotherapy for older adults at risk for suicide: preliminary findings. Prof Psychol Res Pr. (2009) 40:156–64. 10.1037/a0014731 20574546 PMC2889497

[114] Lapierre S Dub,é M Bouffard L Alain M. Addressing suicidal ideations through the realization of meaningful personal goals. Crisis (2007) 28:16–25 10.1027/0227-5910.28.1.16 17555029

[115] Brandtstädter J Rothermund K. Intentional self-development: exploring the interfaces between development, intentionality, and the self. Nebr Symp Motiv. (2002) 48:31–75. 12113000

[116] Hinrichsen GA Hernandez NA. Factors associated with recovery from and relapse into major depressive disorder in the elderly. Am J Psychiatry (1993) 150:1820–5. 10.1176/ajp.150.12.1820 8238636

[117] Chatterton L Hall PL Tarrier N Cognitive therapy for low self-esteem in the treatment of depression in an older adult. Behav and Cogn Psychother. (2007) 35:365–9. 10.1017/S1352465807003608

[118] McLaren S Gomez R Bailey M Van Der Horst RK. The association of depression and sense of belonging with suicidal ideation among older adults: applicability of resiliency models. Suicide Life Threat Behav. (2007) 37:89–102. 10.1521/suli.2007.37.1.89 17397283

[119] Malone KM Oquendo MA Haas GL Ellis SP Li S Mann JJ. Protective factors against suicidal acts in major depression: reasons for living. Am J Psychiatry (2000) 157:1084–8. 10.1176/appi.ajp.157.7.1084 10873915

[120] Snyder CR Rand KL. The case against false hope. Am Psychol. (2003) 58:820–2; authors' reply 823–4 10.1037/0003-066X.58.10.820 14585003

[121] Edwards MJ Holden RR. Coping, meaning in life, and suicidal manifestations: examining gender differences. J Clin Psychol. (2001) 57:1517–34. 10.1002/jclp.1114 11745593

[122] Dervic K Oquendo MA Grunebaum MF Ellis S Burke AK Mann JJ. Religious affiliation and suicide attempt. Am J Psychiatry (2004) 161:2303–8. 10.1176/appi.ajp.161.12.2303 15569904

[123] Farioli Vecchioli S Sacchetti S Nicolis di Robilant V Cutuli D The role of physical exercise and omega-3 fatty acids in depressive illness in the elderly. Curr Neuropharmacol. (2018) 16:308–26. 10.2174/1570159X15666170912113852 28901279 PMC5843982

[124] Wang J Um P Dickerman BA Liu J. Zinc, Magnesium, Selenium and Depression: A Review of the Evidence, Potential Mechanisms and Implications. Nutrients (2018) 10:E584. 10.3390/nu10050584 29747386 PMC5986464

[125] Qian Y Sun L Zhou C Ge D Zhang L. The association between suicidal ideation and sleep quality in elderly individuals: A cross-sectional study in Shandong, China. Psychiatry Res. (2017) 256:453–7. 10.1016/j.psychres.2017.07.017 28709060

[126] McCall WV Benca RM Rosenquist PB Riley MA McCloud L Newman JC. Hypnotic medications and suicide: risk, mechanisms, mitigation, and the FDA. (2017) Am J Psychiatry. 174:18–25. 10.1176/appi.ajp.2016.16030336 27609243 PMC5205566

[127] Bishop TM Simons KV King DA Pigeon WR. Sleep and suicide in older adults: an opportunity for intervention. Clin Ther. (2016) 38:2332–9. 10.1016/j.clinthera.2016.09.015 27751672

[128] O'Rourke N Heisel MJ Canham SL Sixsmith. Predictors of suicide ideation among older adults with bipolar disorder. PLoS ONE (2017) 12:e0187632. 10.1371/journal.pone.0187632 29145409 PMC5690620

[129] Shah A. A replication of a possible relationship between elderly suicide rates and smoking using five-year data on suicide rates? A cross-national study J Inj Violence Res. (2010) 2:35–40. 10.5249/jivr.v2i1.44 21483196 PMC3134894

[130] Knox KL Litts DA Talcott GW Feig JC Caine ED. Risk of suicide and related adverse outcomes after exposure to a suicide prevention programme in the US Air Force: cohort study. BMJ (2003) 327:1376. 10.1136/bmj.327.7428.1376 14670880 PMC292986

[131] Owens C Lambert H Donovan J Lloyd KR. A qualitative study of help seeking and primary care consultation prior to suicide. Br J Gen Pract (2005) 55:503–9. 16004734 PMC1472785

[132] Moskos MA Olson L Halbern SR Gray D. Utah youth suicide study: barriers to mental health treatment for adolescents. Suicide Life Threat Behav. (2007) 37:179–86. 10.1521/suli.2007.37.2.179 17521271

[133] Das JK Salam RA Lassi ZS Khan MN Mahmood W Patel V. Interventions for adolescent mental health: an overview of systematic reviews. J Adolesc Health (2016) 59:S49–S60. 10.1016/j.jadohealth.2016.06.020 27664596 PMC5026677

[134] Motohashi Y Kaneko Y Sasaki H. Community-based suicide prevention program in Japan using a health promotion approach. Environ Health Prev Med. (2004) 9:3–8. 10.1265/ehpm.9.3 21432331 PMC2723381

[135] Gallego JA Rachamallu V Yuen EY Fink S Duque LM Kane JM. Predictors ofsuicide attempts in 3.322 patients with affective disorders and schizophreniaspectrum disorders. Psychiatry Res. (2015) 228:791–6. 10.1016/j.psychres.2015.05.024 26077849 PMC4532595

[136] Pompili M Innamorati M Masotti V Personnè F Lester D Di Vittorio C. Suicide in the elderly: a psychological autopsy study in a North Italy area (1994-2004). Am J Geriatr Psychiatry (2008) 16:727–35. 10.1097/JGP.0b013e318170a6e5 18556398

[137] Madhusoodanan S Ibrahim FA Malik A. Primary prevention in geriatric psychiatry. Ann Clin Psychiatry (2010) 22:249–61. 21180656

[138] Forsman AK Schierenbeck I Wahlbeck K. Psychosocial interventions for the prevention of depression in older adults: systematic review and meta-analysis. J Aging Health (2011) 23:387–416. 10.1177/0898264310378041 20935250

